# A serum biomarker panel and miniarray detection system for tracking disease activity and flare risk in lupus nephritis

**DOI:** 10.3389/fimmu.2025.1541907

**Published:** 2025-05-01

**Authors:** Chenling Tang, Gongjun Tan, Aygun Teymur, Jiechang Guo, Arturo Haces-Garcia, Weihang Zhu, Richard Williams, Jing Ning, Ramesh Saxena, Tianfu Wu

**Affiliations:** ^1^ Department of Biomedical Engineering, University of Houston, Houston, TX, United States; ^2^ Department of Mechanical Engineering Technology, University of Houston, Houston, TX, United States; ^3^ Iolight Co, Hampshire, United Kingdom; ^4^ Department of Biostatistics, University of Texas MD Anderson Cancer Center, Houston, TX, United States; ^5^ Division of Nephrology, University of Texas, Southwestern Medical Center, Dallas, TX, United States

**Keywords:** biomarker panel, lupus nephritis, disease monitoring, flare assessment, point-of-care diagnostics

## Abstract

**Introduction:**

Lupus nephritis (LN) leads to end stage renal disease (ESRD), and early diagnosis and disease monitoring of LN could significantly reduce the risk. however, there is not such a system clinically. In this study we aim to develop a biomarker-panel based point-of-care system for LN.

**Methods:**

Immunoassay screening combined with genomic expression databases and machine learning techniques was used to identify a biomarker panel of LN. A quantitative biomarker-panel mini-array (BPMA) system was developed and the sensitivity, specificity, reproducibility, and stability of the were examined. The performance of BPMA in disease monitoring was validated with machine models using a larger cohort of LN. The BPMA was also used to determine LN flare using a machine-learning generated flare score (F-Score).

**Results:**

Among 32 promising LN serum biomarkers, VSIG4, TNFRSF1b, VCAM1, ALCAM, OPN, and IgG anti-dsDNA antibody were selected to constitute an LN biomarker Panel, which exhibited excellent discriminative value in distinguishing LN from healthy controls (AUC = 1.0) and active LN from inactive LN (AUC = 0.92), respectively. Also, the 6-biomarker panel exhibited a strong correlation with key clinical parameters of LN. A multiplexed immunoarray was constructed with the 6-biomarker panel (named BPMA-S6 thereafter). An LN-specific 8-point standard curve was generated for each protein biomarker. Cross-reaction between these biomarkers was minimal (< 1%). BPMA-S6 test results were highly correlated with those from ELISA (Spearman’s correlation: fluorescent detection, rs = 0.95; colorimetric detection, rs = 0.91). The discriminative value of BPMA-S6 for LN was further validated using an independent cohort (AUC = 0.94). Using a longitudinal cohort of LN, the derived F-Score exhibited superior discriminative value in the training dataset (AUC = 0.92) and testing dataset (AUC=0.82) to distinguish flare vs remission.

**Conclusion:**

BPMA-S6 may represent a promising point-of-care test (POCT) for the diagnosis, disease monitoring, and assessment of LN flare.

## Introduction

Systemic lupus erythematosus (SLE) is a multifactorial autoimmune disease with high comorbidity and multiorgan damage (1). Lupus nephritis (LN), one of the most severe organ manifestations of SLE, affects 30%–60% of adults and up to 70% of children with SLE disease (2). Fifteen years after the first diagnosis, 10%–30% of LN patients will deteriorate into end-stage renal disease (ESRD), requiring regular hemodialysis or kidney transplantation (3). Hence, LN flare represents a significant risk for SLE patients and may cause a tremendous socioeconomic burden. For effective clinical management of LN flare, diagnosis, disease monitoring, and LN flare assessment are critical. The gold standard for the clinical diagnosis of LN is a renal biopsy (4); however, it is invasive and may cause further kidney damage (5). Hence, it is particularly unfeasible to perform multiple renal biopsies to monitor disease progression or drug responses for LN (6). Compared to the traditional renal biopsy approach, the detection of LN-specific biomarkers in body fluids could be minimally invasive or noninvasive for early diagnosis and disease monitoring of LN (7). It is especially attractive and useful when serial detection or real-time monitoring of LN is needed (8). Importantly, the detection of urine or serum-based biomarkers for LN has great potential to inform the disease activity of LN using simple but rapid tests (1, 9). Currently, the need for biomarker-based tests is underscored by the inadequacy of conventional clinical measures to detect ongoing disease activity in lupus kidneys and early recurrence of nephritis (10). Given the heterogeneity of LN, it is particularly difficult to accurately diagnose or monitor LN or predict LN flare using a single biomarker. The up-and-coming Omics technologies, bioinformatics, and machine learning techniques may be promising in identifying a disease-specific biomarker panel to improve the sensitivity and specificity for early diagnosis and disease monitoring of LN (11–14).

Current clinical biomarker tests, such as polymerase chain reaction (PCR), biosensor, chemical reaction-based assays, enzyme-linked immunosorbent assay (ELISA), and lateral flow immunoassay (LFA), are mostly designed for the detection of individual biomarkers, such as DNA/RNA, blood glucose, electrolytes, enzymes, hormones, lipids, other metabolites, and proteins (15). These assays are either qualitative or quantitative and have been instrumental in detecting individual biomarkers. However, these platforms cannot simultaneously detect multiple biomarkers as a biomarker panel due to the technical difficulty of multiplexing.

Currently, commercially available multiplex assays include bead-based arrays such as Luminex^®^ xMAP^®^ (Luminex Corporation, Austin, TX) and planar microarrays such as Kiloplex array (Raybiotech, Inc, Peachtree Corners, GA). The bead-based arrays immobilize capture molecules on the surface of the bead to achieve multiplexing, whereas the planar microarray uses the arrayed capture molecules for the detection of multiple targets on a chip (16). However, these technologies not only need specialized large laboratory equipment to run the experiments and detect the signals but also need sophisticated commercial software for data analysis.

In this study, we aimed to (1) identify a biomarker panel that can reflect the disease activity of LN with superior sensitivity and specificity; (2) to develop a low-cost, quantitative, portable, and multiplexed biomarker panel miniarray (BPMA) system, which requires a smaller reaction volume, shorter incubation time, and an easy-to-use 3D-printed chip cassette for sample loading and washing, a low-cost microscopic array reader for smartphone imaging and a user-friendly open-source smartphone app for data analysis and reporting. More importantly, this may represent the first detection system for quantifying autoantibody (anti-dsDNA), protein biomarkers, and immune complexes on one chip. All these features could be beneficial for future diagnosis and disease monitoring of LN in homecare or community medicine.

## Materials and methods

### Selection of serum biomarkers of LN from the literature

Based on our recent literature analysis on biomarkers in LN and SLE (13), 255 relevant research articles were retrieved from PubMed, and candidate biomarkers exhibiting an overall AUC > 0.8 with *p*-value < 0.05, and sample size > 10 per group were selected. After screening for the availability of properly paired antibodies (capture antibody and detection antibody), 32 promising LN biomarkers were selected for independent validation (**Supplementary Table S1**). Additionally, a novel high-performance candidate LN biomarker, V-set, and immunoglobulin domain containing 4 (VSIG4), recently discovered by our group (17), as well as IgG anti-dsDNA were incorporated into our initial biomarker panel for validations.

### Patients and clinical samples

Serum samples from lupus nephritis and healthy controls were collected at the University of Texas, Southwestern Medical Center in Dallas. All human subject-related procedures were performed in accordance with the University of Houston-approved IRB protocols, and informed consent was obtained from all subjects before sample collection. Detailed demographics and clinical information are summarized in **Table 1**. Three different cohorts were used for biomarker panel discovery, disease diagnostics, and monitoring and flare assessment, respectively. All human serum samples were aliquoted upon receipt and stored at − 80°C. The active lupus nephritis (LN-Active) patient group was defined as Systemic Lupus Erythematosus Disease Activity Index (SLEDAI) greater than 4 and the renal domain of SLEDAI (rSLEDAI) greater than 0. The inactive lupus nephritis (LN-Inactive) patient group was defined as having a SLEDAI less than 4 and rSLEDAI equal to 0. The lupus nephritis flare (LN-Flare) patient group was defined with the following three criteria: (1) the urine protein/creatinine ratio (uPCR) > 0.5 mg/mg (2) serum creatinine increase > 15%–20% of the baseline (3) > 5 RBC/HPF in urine. Otherwise, the patient was defined as being in remission lupus nephritis (LN-Remission) (18). The matched healthy controls or disease controls were tested alongside the lupus nephritis samples.

**Table 1:** Demographic and clinical characteristics of subjects.

		LN-Active	LN-Inactive	Mann–Whitney p-value
Cohort 1: Lupus nephritis cross-sectional patients				
Total No. of subjects		49	13	–
Female (No.; %)		89.80%	100.00%	–
Age (mean ± SE, years)		28.90 ± 8.47	34.51 ± 13.14	0.053
Ethnicity (Asian/Black/Hispanic/White, no)		4/23/12/20	1/1/0/11	–
SLEDAI (median; interquartile)		9 (8–12)	2 (0–4)	< 0.0001
Renal SLEDAI (median; interquartile)		4 (4–8)	0 (0–0)	< 0.0001
No. of patients with renal SLEDAI = 0 (%)		0.00%	100%	–
AI (median; interquartile)		5 (2.0–8.0)	4 (2.5–4.5)	0.49
CI (median; interquartile)		4 (2.0–6.0)	1 (0.5–2.0)	0.067
Serum creatinine (mg/dl, median; interquartile)		0.920 (0.71–1.84)	0.75 (0.64–0.81)	0.058
Urine protein:creatinine ratio (mg/mg, median; interquartile)		2.91 (1.43–5.31)	0.20 (0.18–0.30)	< 0.0001
Cohort 1: Healthy controls				
Total No. of subjects		23		
Female (No.; %)		65.22%		
Age (mean ± SE; years)		31.33 ± 10.88		
Ethnicity (Asian/Black/Hispanic/White, no)		5/10/0/8		
Cohort 2: Lupus nephritis cross-sectional patients				
Total No. of subjects	36		19	–
Female (No.; %)	86.11%		84.21%	–
Age (mean ± SE; years)	31.90 ± 10.25		39.67 ± 14.30	0.032
Ethnicity (Asian/Black/Hispanic/White, no)	12/8/9/7		6/1/4/8	–
SLEDAI (median; interquartile)	10 (6–12)		2 (0–2.5)	< 0.0001
Renal SLEDAI (median; interquartile)	4 (4–8)		0 (0–0)	< 0.0001
No. of patients with renal SLEDAI = 0 (%)	12.82%		100%	–
No. of patients with DNA positive (%)	71.70%		32.25%	–
Serum creatinine (mg/dl, median; interquartile)	0.93 (0.72–1.48)		0.87 (0.67–1.08)	0.512
Urine protein:creatinine ratio (mg/mg, median; interquartile)	1.92 (1.13–3.31)		0.29 (0.18–0.47)	< 0.0001
Cohort 2: Chronic kidney disease patients				
Total No. of subjects	11			
Female (No.; %)	63.64%			
Age (mean ± SE; years)	44.92 ± 14.42			
Ethnicity (Asian/Black/Hispanic/White, no)	0/6/1/4			
Serum creatinine (mg/dl, median; interquartile)	1.63 (1–2)			
Urine protein:creatinine ratio (mg/mg, median; interquartile)	3.7 (1.44–6.35)			
CKD stage (2/3/4/5, no)	3/3/3/2			
Cohort 2: Health control				
Total No. of subjects	23			
Female (No.; %)	69.56%			
Age (mean ± SE; years)	32.53 ± 10.27			
Ethnicity (Asian/Black/Hispanic/White, no)	2/12/0/9			
Cohort 3: Lupus nephritis longitudinal patients				
Total No. of patients		8	7	–
Total No. of subjects		33	18	–
Average visit interval (month)		11.12	5.57	–
Female (No.; %)		100.00%	71.43%	–
Age at baseline (mean ± SE; years)		31.90 ± 12.45	26.45 ± 6.63	0.77
Ethnicity (Asian/Black/Hispanic/White, no)		1/2/0/5	2/3/0/2	–
SLEDAI (median; interquartile)		5 (4–8)	8 (4.6–11)	1.50*E*−01
Renal SLEDAI (median; interquartile)		4 (0–4)	4 (1–8)	1.20*E*−01
No. flare status at visit (%)		63.64%	67%	–
Serum creatinine (mg/dl, median; interquartile)		0.76 (0.70–0.83)	0.81 (0.72–0.95)	0.52
Urine protein:creatinine ratio (mg/mg, median; interquartile)		0.7 (0.30–1.40)	0.62 (0.28–2.74)	5.70*E*−01

### Measurement of serum biomarkers using ELISA

In the prescreening stage, each of the 32 protein biomarkers (**Supplementary Table S1**) was measured individually using ELISA, assembled with paired capture antibodies (cAbs) and detection antibodies (dAbs) in LN and healthy control (HC) samples, where various serum dilutions were optimized. The list of analytes and manufacturers is provided in **Supplementary Table S1**. Of these, nine markers showed no difference between LN and HC groups, and another seven markers were below the lowest limit of detection (L-LOD) in the serum samples. The remaining 16 biomarkers with elevated expression in the LN group were further validated, along with IgG anti-dsDNA, using commercial or in-house ELISA kits and a cohort of 85 serum samples (LN-Active, *N* = 49; LN-Inactive, *N* = 13; healthy control, *N* = 23). ELISA signals were read using an Epoch plate reader (BioTek, VT, Winooski, Vermont) at 450 nm. In addition to IgG anti-dsDNA, the serum concentrations of the remaining biomarkers were determined by the four-parameter logistic (4PL) standard curves based on serially diluted standards.

### Development of biomarker-panel miniarray (BPMA-S6)

The LN biomarker panel (BPMA-S6) consists of IgG anti-dsDNA as well as five candidate protein biomarkers—VSIG4, Tumor Necrosis Factor Receptor 2 (TNFRSF1B), Vascular Cell Adhesion Molecule 1 (VCAM1), activated leukocyte cell adhesion molecule (ALCAM) and Osteopontin (OPN)—selected based on (1) statistical analysis of our screening test data, (2) analysis of lupus-related Omics databases, and (3) machine-learning algorithms. The epoxy-modified polymer slide (STRATEC Consumables GmbH, Birkenfeld, Germany) was chosen for cAb/antigen immobilization and subsequent detection, based on our previous work (19–21). Capture antibodies against VSIG4, TNFRSF1B, VCAM1, ALCAM, and OPN (R&D Systems, Minneapolis, MN) were individually diluted to the optimal concentrations (**Supplementary Table S2**) in 1× phosphate-buffered saline (PBS) buffer (R&D Systems), and dsDNA (Sigma, St. Louis, MO) was diluted to 200 μg/ml with 100 μg/ml mBSA buffer (Sigma, St. Louis, MO). A 100-μg/ml BSA-Biotin solution (Thermo Fisher Scientific, Waltham, MA) and 1× PBS buffer (R&D Systems) were used as positive and negative controls, respectively. All diluted antigen/antibody and control solutions were then transferred to a 384-well microtiter plate (Thermo Fisher Scientific) and briefly centrifuged (2,000×*g* at 4°C for 2 min) to remove aggregates and bubbles. The highest standard (Standard 1 [STD1]) mixture and detection antibody cocktail were prepared as indicated in **Supplementary Table S2**. The serum concentrations of the five protein biomarkers were calculated using the 4PL standard curves generated from serially diluted standards derived from STD1.

**Figure 1:**
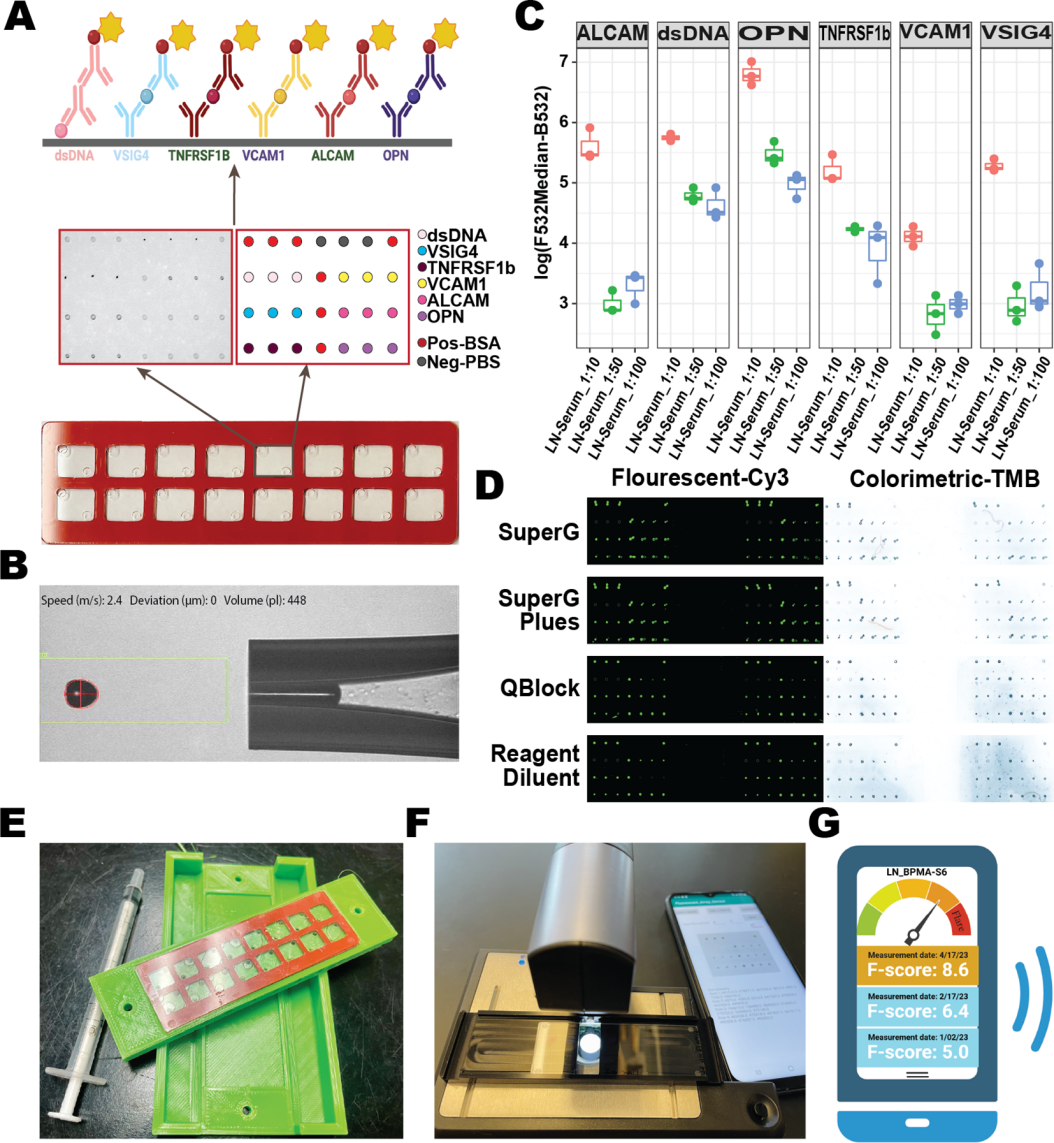
Construction and optimization of the lupus biomarker-panel miniarray (BPMA-S6). **(A)** Schematic of the slide construction for BPMA-S6. **(B)** Active control image of antibody/antigen contactless spotting. **(C)** Boxplot of signal intensities across different serum dilution ratios. **(D)** Array images showing the performance buffers in both fluorescent and colorimetric groups. **(E)** Prototype of the 3D-printed BPMA washer. **(F)** Customized portable colorimetric microscope. **(G)** Smartphone application developed for BPMA-S6.

The slides were loaded onto a noncontact microarray printing robot (sciFLEXARRAYER S3; SCIENION GmbH, Berlin, Germany), and each biomarker was printed in triplicates (drop volume: 450 pl ± 20 pl) on the slide using PDC90 (SCIENION) at 25°C and 60% humidity (**Figure 1B**). Positive and negative controls were printed in a nonsymmetrical “T” shape to indicate the orientation of each array, as well as the positions of each row and column (**Figure 1A**). After printing, the slides were dried in the printing chamber overnight and assembled with SecureSeal™ Hybridization 16-well Chambers (Grace Bio-Labs, Bend, OR). The slides were stored in a vacuum-sealed bag with desiccant and stored at 4°C or − 20°C prior to use.

### Quantitative detection of biomarkers in LN serum with BPMA-S6

The BPMA-S6 slides were brought to room temperature at least 30 min before testing. Two BSA-based dilution buffers—reagent diluent (R&D System) and QBLOCK™ (Grace Bio-Labs)—and two non-BSA dilution buffers—Super G™ (Grace Bio-Labs) and Super G™ plus (Grace Bio-Labs)—were used to dilute the serum samples or standard mixtures, respectively. The slides were blocked with 40 μl of dilution buffer per subarray by injecting it through the SecureSeal chamber input inlet. Serum samples and standard mixtures (seven twofold diluted mixtures) were diluted with dilution buffer, and 40 μl of each solution was injected into each subarray and incubated on a shaker at room temperature for 2 h, followed by three washes with TBS-Tween 20. The array was then incubated with 40 μl of a cocktail dAb solution in each array at room temperature on a shaker for 1 h. Slides were washed three times, followed by incubation with 40 μl streptavidin-HRP solution for 30 min. The slides were washed again before incubation with 40 μl of Cy™3 streptavidin (Jackson ImmunoResearch, West Grove, PA) for fluorescent signals or SeramunBlau (Seramun Diagnostica GmbH, Heidesee, Germany) solution for colorimetric signals. Finally, the slides were washed three times, and the gasket was detached from the slide. After rinsing with deionized water and air drying, the slide was scanned using a GenePix 4000B scanner (Molecular Devices, San Jose, CA) for fluorescent signals, an office scanner (Epson, Los Alamitos, CA), or a customized portable microscopic reader (Iolight Limited, UK, Hampshire, United Kingdom) for colorimetric signals. Each spot was quantified as described previously (19, 22, 23).

### Gene expression analysis of promising biomarker candidates

Six publicly available lupus microarray databases (**Supplementary Table S3**) were downloaded to crossvalidate the transcription expression levels in different tissues or cell types. Each dataset was normalized and scaled before comparisons between lupus and healthy groups were made, using average fold-change and the Wilcoxon test. The aggregated, clustered “h5ad” file of lupus PBMC single-cell RNA-seq (scRNA-seq) data was downloaded from the GEO Database (GSE174188) (24). A total of 1.2 million PBMC cells from 162 lupus patients and 99 healthy control patients were analyzed with Scanpy 1.9 to examine biomarker expression levels across different clusters and deduce potential cell origin (25).

### Construction of the PPI network

The PPI network was constructed by inputting the protein list into the Search Tool for the Retrieval of Interacting Genes (STRING) (26), using the full STRING network and interaction score > 0.15. Cytoscape and its plug-in Cyto-Hubba were then utilized to calculate the degree of each protein to identify the hub gene based on its Maximal Clique Centrality (MCC) rank (27, 28).

### Flare-Score based on the BPMA-S6 measurement

The BPMA Flare-Score was derived with reference to previous algorithms for multibiomarker disease activity scores, with few modifications (29–31). In our algorithm, the serum concentrations of five protein biomarkers and the binary values of anti-dsDNA antibody (“1” for “positive” and “− 1” for “negative”) were used to train the Least Absolute Shrinkage and Selection Operator (LASSO) to optimize a generalized linear model for assessing Flare and Remission status of LN. The Flare-Score (*F*-Score) was generated as the sum of each biomarker serum level multiplied by the respective weights:


$$\begin{array}{l} F-\text{Score}=6.79-7.20\cdot \text{ALCAM }\cdot 10^{-5}-3.16\cdot \text{dsDNA}\cdot 10^{-1}\\ +1.37\cdot \text{OPN}\cdot 10^{-4}-1.41\cdot TNFRSF1B\cdot 10^{-4}\\ +4.00\cdot VCAM1\cdot 10^{-6}+7.99\cdot VSIG4\cdot 10^{-4} \end{array}$$

In the training step, the LASSO-Flare model was fine-tuned with 33 longitudinal samples from eight LN patients using threefold crossvalidation to determine the optimal regularization strength (lambda) using the “glmnet” package (32). Next, the same set of weights was used to calculate the *F*-Score for an independent testing dataset of 18 longitudinal samples from seven LN patients. The optimal cut-point was determined by maximizing the Youden index using the “cutpointr” package (33).

### 3D-printed BPMA chip cassette

**Figure 2:**
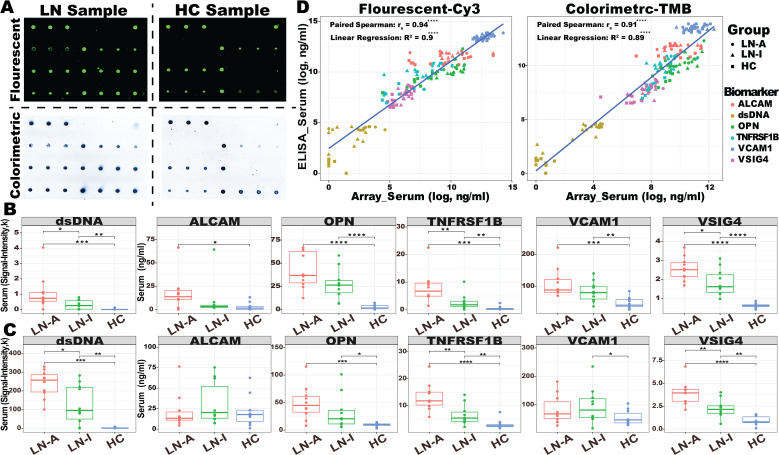
Assaying 30 serum samples with BPMA-S6. **(A)** Representative BPMA array images in fluorescent and colorimetric settings using serum samples from LN and HC. **(B, C)** A total of 30 serum samples from active LN (LN-A, *N* = 10, red), inactive LN (LN-I, *N* = 10, green), and healthy controls (HC, *N* = 10, blue) were tested using BPMA fluorescent **(B)** and colorimetric **(C)** signals. **(D)** Linear regression and Spearman’s correlation analyses were performed to assess the correlation between BPMA and ELISA measurements. Asterisks indicate the level of statistical significance: *n.s. p* > 0.05; ^*^ *p* < 0.05; ^**^ *p* < 0.01; ^***^ *p* < 0.001; ^****^ *p* < 0.0001.

In order to suit BPMA for easy-to-use at home or community settings, we designed an all-in-one chip cassette for sample loading and washing. The cassette was designed in SolidWorks software (3DS) and manufactured using a 3D printer (Qidi Max X) with PLA plastic materials. The chip cassette was optimized through several design iterations to achieve the following goals: (1) the top openings of the cassette align exactly with the corresponding inlet on each subarray for the entry of samples, reagents, or washing buffer; (2) the side openings collect the waste from the outlet of each subarray; (3) the chip cassette is airtight, and each subarray can be washed separately with the aid of a syringe. The injection topping (length:width:height: 105.8:29.5:29.5 mm) and bottom tray (length:width:height: 105.8:59.5:11 mm) were printed separately, and BPMA chip can be inserted between the injection topping and the bottom tray (**Figure 2E**; **Supplementary Figure S1**). The topping injection holes were designed into a cone shape (diameters of 5.2 mm at the top and 1.6 mm at the bottom) to fit 1~5 ml syringes for adding serum or reagents or washing buffer. In the bottom tray, the outlet holes are connected through the channel (4 mm in diameter) to a waste tank which was designed to collect the waste.

### Customized BPMA imaginer

The microscopic imager was built starting from an ioLight 2-mm portable microscope (https://iolight.co.uk/product/portable-microscope-x150-2mm-field-of-view/). The lens was removed and replaced with a 10-mm focal length lens. This lens was mounted in the microscope focusing motor using a custom-made 3D printed nylon mounting ring and a 2.5-mm diameter aperture was inserted in front of the lens to restrict its aperture of the lens to increase image contrast. The mount for the image sensor in the microscope was modified so that the sensor was positioned at the correct distance from the lens to deliver an image with a horizontal field of view of 4.8 mm. To accommodate this wider field of view, the diameter of the substage illuminator was increased from 7 to 10 mm. The microscope can be controlled via a computer or a smartphone, which views live images and saves them. This interface is accomplished by a web server running on the microscope so that a web browser can be used to control the microscope and view images. This web server was modified to enable a monochrome imaging option. The cost of this imager is around $100 in a large volume manufacturing.

### Statistical analysis

All data were analyzed, plotted, and visualized using the R 4.1.0 language and the “ggplot2” package (34). Group significant differences were determined using the Kruskal–Wallis test and group-wise comparisons of statistical significance (*p*-values) were performed using the Wilcoxon test unless stated otherwise. AUC analysis was performed using a pROC package (35), and LASSO analysis was performed with the “glmnet” package (32). The correlation between biomarker levels and clinical or pathological parameters was determined using Spearman’s correlation coefficient and the COR package (36), and their interchangeable relationship was measured using a linear regression model. The coefficient of variation (CV) is calculated as the mean divided by the standard deviation of a set of measurements. The limit-of-detection (LOD) is the lowest concentration, calculated from the standard curve using the mean of the blanks plus three times the blanks’ standard deviation. Six machine-learning models were trained with “SciKit-learn” and plotted with “matplotlib” from Python (37).

## Results

### Selection of serum biomarker panel for LN

**Figure 3:**
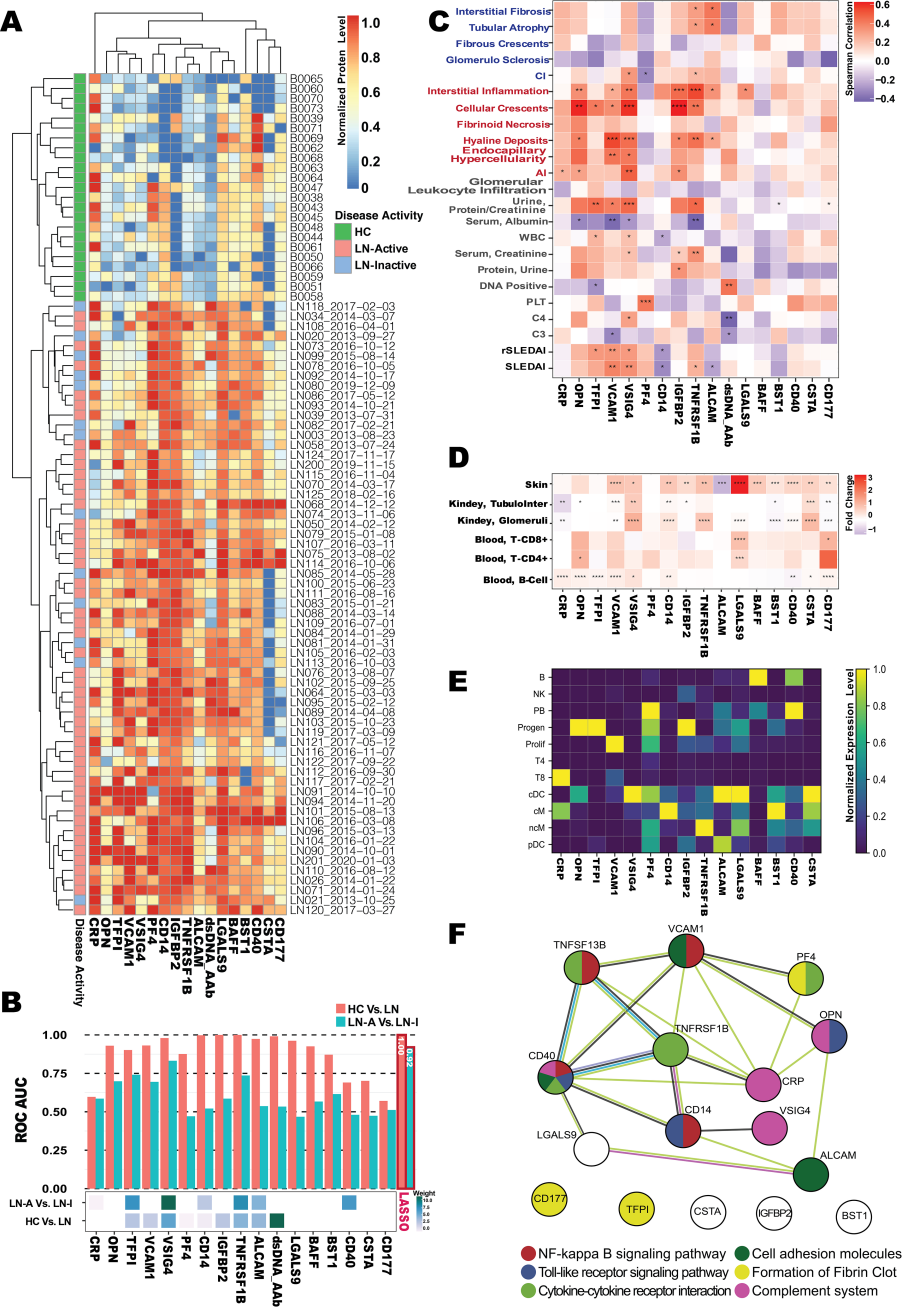
Omics selection for the LN serum biomarker panel. **(A)** Heatmap of the ELISA-based normalized protein levels for 17 promising LN serum biomarkers from LN-Active (*N* = 49), LN-Inactive (*N* = 13), and healthy control (*N* = 23) groups, with row clustering performed using Euclidean distance. **(B)** Barplot of AUC values (upper) for individual biomarkers distinguishing LN from HC, or LN-Active (LN-A) from LN-Inactive (LN-I), and heatmap of the Least Absolute Selection Shrinkage Operator (LASSO) weight scores (lower) for the biomarker panel distinguishing LN from HC, or LN-A from LN-I. **(C)** Correlation plot of serum biomarker levels with clinical parameters, where the color of each square represents Spearman’s correlation coefficient value, and significance levels are indicated with asterisks. **(D)** Gene expression profiles of validated biomarkers at six tissue-specific bulk-seq databases, with square colors representing fold-changes between lupus and HC, and significance levels indicated with asterisks. **(E)** Potential cellular origins of the selected biomarkers were identified using a lupus scRNA database comprising 1.2 million PBMCs. The color of each square represents the normalized expression level. **(F)** Protein–protein interaction (PPI) network among the 17 promising biomarkers constructed using the STRING database, where node colors indicate the associated pathways and edge colors represent predicted functional associations: yellow for text-mining evidence, black for coexpression evidence, purple for experimental evidence, and blue for sequence similarity evidence. ^*^ *p* < 0.05; ^**^ *p* < 0.01; ^***^ *p* < 0.001; ^****^ *p* < 0.0001.

Serum samples from an independent cohort of 85 subjects (49 LN-Active, 13 LN-Inactive, and 23 HC) were used in validation studies of 17 promising biomarkers using sandwich ELISA, as shown in **Figure 3A**. Ten of the 17 biomarkers exhibited outstanding discriminatory abilities (AUC ≥ 0.9) in distinguishing LN patients from healthy controls. Interestingly, TNFRSF1B, TFPI, OPN, VCAM1, and VSIG4 showed strong discriminatory abilities (AUC ≥ 0.7) in differentiating LN-Active from the LN-Inactive group, as shown in **Figure 3B**. Importantly, when six biomarkers—IgG anti-dsDNA, TNFRSF1B, TFPI, OPN, VCAM, and VSIG4—were integrated into a single biomarker panel using the LASSO model, IgG anti-dsDNA and VSIG4 were found to have the highest weights contributing to the discriminatory capability between HC vs. LN and LN-Active vs. LN-Inactive, respectively (**Figure 3B**). Next, we investigated which biomarkers were associated with clinical (e.g., SLEDAI, rSLEDAI, C4, and protein urine/creatinine) or renal pathological parameters (e.g., renal activity index [AI] and renal chronicity index [CI], and their components). As shown in **Figure 3C**, VCAM1, and VSIG4 exhibited a significant positive correlation with both SLEDAI and rSLEDAI, whereas the CD14 displayed a significant negative correlation with them. TNFRSF1B, VSIG4, OPN, IGFBP2, and VCAM1 all showed sound correlations with AI and its components. Unsurprisingly, the anti-dsDNA antibody results measured by in-house ELISA were consistent with the clinical testing data of anti-DNA. To examine the functional aspects and cellular origins of these protein biomarkers at the transcriptional level, particularly in relation to LN, six lupus tissue-specific RNA expression databases and an LN PBMC single-cell RNA sequencing (scRNA-Seq) dataset comprising 1.2 million cells were queried against the validated protein biomarkers (**Figures 3D, E**). The unique transcription pattern of each biomarker and the underlying molecular mechanisms may reflect the heterogeneity of lupus and subsequent LN. For instance, VCAM1 was found to be relatively highly expressed in the CD8+ T-cell cluster, while ALCAM was highly expressed in conventional and plasmacytoid dendritic cells (cDC and pDC). In the protein–protein interaction (PPI) network analysis (**Figure 3F**), six lupus-related pathways were selected to construct the biomarker panel based on their molecular functions and associations with lupus and LN (38–43).

The following six criteria were used to construct a biomarker panel for the diagnosis or disease monitoring of LN on the BPMA chip: (1) the ability to discriminate LN from HC (AUC > 0.9) and, more importantly, LN-Active from LN-Inactive (AUC > 0.7); (2) a significant positive correlation with key clinical/pathological parameters *(r_s_* > 0 and *p* ≤ 0.05); (3) significant differential gene/protein expression levels in SLE tissues (fold change > 1.5 and *p* ≤ 0.05); (4) gene/protein expression originating from different PBMC cell types; (5) involvement in six predefined disease-related molecular and biological pathways; and (6) robust performance on the BPMA chip (i.e., producing detectable signal, with signal intensity correlating with analyte concentration).

Finally, the BPMA-S6 biomarker panel includes IgG anti-dsDNA, VSIG4, TNFRSF1B, VCAM1, ALCAM, and OPN.

### Development of BPMA-S6 immunochip for POC

The optimized concentration of antibody/antigen (**Supplementary Table S2**) was used to create a sandwich-structure array for the simultaneous detection of five protein biomarkers—VSIG4, TNFRSF1B, VCAM1, ALCAM, and OPN—as well as IgG anti-dsDNA autoantibody. The “positivity” derived from our BPMA-based anti-dsDNA assay correlated with clinical test results of anti-dsDNA antibody (AUC = 0.75), with a specificity of 95% and sensitivity of 48% (**Supplementary Figures S9A, B**). To achieve the POC purpose, a SecureSeal™ Hybridization 16-well Chamber was used, resulting in a higher signal-to-noise ratio and a reduced reaction volume (from 100 to 40 µL) compared to a traditional gasket (**Figure 1A**). Moreover, it minimized the contamination risk during the incubation and washing steps. Given that the 1:10 serum dilution ratio produced the strongest signal across all biomarkers and the lowest spot signal variations (spot signal CV: 17.45%, 19.78%, and 28.54% for 1:10, 1:50, and 1:100 serum dilutions, respectively) (**Figure 1C**), we decided to use the 1:10 serum dilution for subsequent BPMA-S6 assays. For both fluorescent and colorimetric detection, the non-BSA-based SuperG was selected for blocking and serum dilution, after comparing the background noise levels and data quality among four diluent buffers (**Figure 1D**; **Supplementary Figure S2**). In addition, a prototype of a 3D-printed BPMA chip cassette (design and dimensions shown in **Supplementary Figure S1**) and a customized microscopic BPMA reader were successfully developed in this study for the POC application of our BPMA system. It is also worth noting that our recently developed smartphone application for array spot detection and analysis (23) is fully compatible with this BPMA system. Importantly, our smartphone-based imaging and analytical capability (using the microscopic imager and a modified smartphone app) is comparable to that of the laboratory desktop scanner, with a strong correlation between the data obtained from the two devices (Spearman’s correlation *r_s_* = 0.85) (**Supplementary Figure S3**). Taken together, our in-house developed integrated BPMA device is portable and cost-effective, enabling biomarker panel measurement, image acquisition, data analysis, and data reporting within a single system (**Figures 1E–G**). This makes it promising for homecare applications, eliminating the need for large equipment and sophisticated techniques to run the assays.

### Assessment of the panel quantification capability, limit of detection, crossreactivity in multiplex, shelf life, and thermostability of the BPMA-S6 chip

To assess the quantification capability of the BPMA-S6, a serially diluted standard mixture, and a blank control were loaded onto the BPMA-S6 chip, followed by either Cy3-based fluorescence detection or SeramunBlau-based colorimetric detection. The scanned images for both methods are illustrated in **Figure 2A**. Since no human IgG anti-dsDNA antibody standard is available, we used signal intensity as a readout for IgG anti-dsDNA. The standard curves and LOD for each biomarker are shown in **Supplementary Figure S4** and **Supplementary Table S4**. Both fluorescence-based and colorimetric detection methods generated robust, biomarker concentration-dependent standard curves for the quantitative analysis of serum biomarker concentrations in LN samples.

To determine potential crossreactivity between the biomarkers in the multiplex system, the precoated subarrays harboring the six biomarker targets were incubated separately with a single biomarker per subarray, followed by detection with a cocktail dAb solution. As shown in **Supplementary Figure S10B** and **Supplementary Figure S5**, the detection of each biomarker is highly specific with none-to-minimal crossreactivity with other biomarkers (< 1%).

The BPMA-S6 detection time was primarily determined by the serum and detection antibody incubation times during the reaction. Different combinations of four serum incubation times (10, 30, 60, and 120 min) and three detection antibody incubation times (10, 30, and 60 min) were individually evaluated, with their colorimetric array images shown in **Supplementary Figure S10C**. Notably, shortening the incubation time down to 10 min still resulted in good BPMA signals using the colorimetric method (**Supplementary Figure S6**).

The reproducibility of our BPMA-S6 system was evaluated through the following experiments: (1) duplicate testing of three serum samples on the same BPMA-S6 chip (intrachip, CV: 6.87%); (2) testing of three serum samples on three different BPMA-S6 chips (interchip, CV: 14.68%); and (3) testing of three serum samples on three BPMA-S6 chips by three different users (different users, CV: 21.57%). These results indicate that the BPMA-S6 system can generate reproducible results (**Supplementary Figure S7**). Next, we assessed whether the shelf life of the chips (1, 3, and 5 weeks; **Supplementary Figure S10D**) or the storage temperature (− 20°C, 4°C, or room temperature/~ 22°C; **Supplementary Figure S10E**) affected the performance of the BPMA-S6. Our results demonstrated that the BPMA-S6 maintained stable performance for at least 5 weeks at 4°C and for up to 10 days at room temperature.

### Quantitative assay of human serum with BPMA-S6

Using both conventional individual ELISA kits and the BPMA-S6 platform, we measured six biomarkers in a cohort consisting of 10 LN-Active, 10 LN-Inactive, and 10 healthy control serum samples. As shown in **Figure 2A**, biomarker levels/signals in the BPMA-S6 panel were markedly elevated in LN patients compared to healthy controls. A strong correlation between the multiplex array test and individual ELISA results was observed (paired Spearman’s *r_s_* > 0.9) (**Figure 2D**), highlighting the diagnostic potential of the BPMA-S6 system. Additionally, the group-wise differences observed with fluorescent and colorimetric detection methods were highly consistent (**Figures 2B, C**). Group significant difference was determined using the Wilcoxon test (**Figure 2D**) Linear regression and paired Spearman’s correlation analyses comparing ELISA-measured levels of the six biomarkers vs. BPMA-measured levels using fluorescent (left) and colorimetric (right) signaling. Asterisks indicate statistical significance: *n.s. p* > 0.05; ^*^ *p* < 0.05; ^**^ *p* < 0.01; ^***^ *p* < 0.001; ^****^ *p* < 0.0001.

Except for ALCAM in the colorimetric group, all other biomarkers were elevated in the LN group compared to HC. VSIG4, TNFRSF1B, and dsDNA antibodies were significantly higher in the LN-Active group than in the LN-Inactive group in both fluorescent and colorimetric settings. The use of a high-affinity antihuman IgG antibody enabled the detection of anti-dsDNA autoantibodies, as well as immune complexes (ICx) formed with the other five autoantigens (**Figure 2A**) (44, 45). The heatmap in **Supplementary Figure S8** compares the results of the full-set cocktail detection solution (containing both antihuman IgG and the five antigen dAbs), the five antigen dAb only (dAb-Only), and anti-human IgG only (IgG-Only) across 30 serum samples. The results reveal that the full-set detection group is highly correlated with the dAb-Only group (*r_s_* = 0.71, *p*-value = 2.2*e*−16), rather than the IgG-Only group (*r_s_* = 0.30, *p*-value = 1.6*e*−4), indicating that the BPMA-S6 primarily detects free-form autoantigens rather than immune complex-bound forms.

### BPMA-S6 in disease diagnostics and LN monitoring

**Figure 4:**
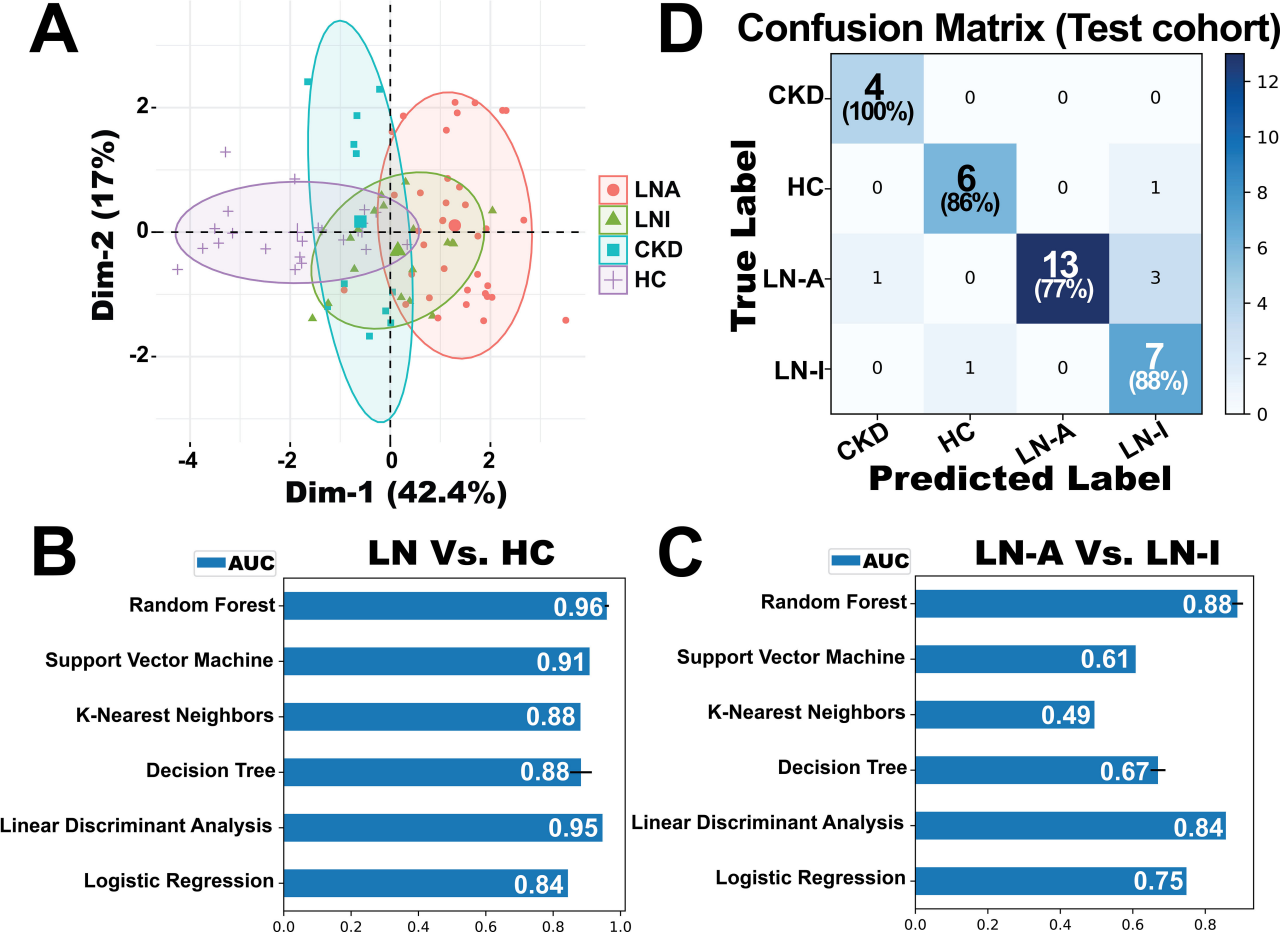
Evaluation of the BPMA-S6 for disease diagnostics and monitoring. **(A)** Principal component analysis (PCA) of a cross-sectional cohort of 89 subjects, including 36 with active lupus nephritis (LN-Active, red), 19 with inactive lupus nephritis (LN-Inactive, green), 11 with chronic kidney disease (CKD, blue), and 23 healthy controls (HC, purple). **(B, C)** Six machine-learning models based on different learning principles were trained using threefold 100-iteration cross-validation to address binary classification problems: LN vs. HC **(B)** and LN-Active vs. LN-Inactive **(C)**. **(D)** Confusion matrix of a fine-tuned multiclass classification Random Forest model evaluated on a testing dataset of 36 samples. The model was trained using 60% (53 samples) of the data for four-group classification.

The diagnostic and disease-monitoring capabilities of BPMA-S6 were evaluated using a cross-sectional cohort of 89 subjects, including 36 with LN-Active, 19 with LN-Inactive, 11 with chronic kidney disease (CKD), and 23 HC. Principal component analysis (PCA), a linear dimensionality reduction method, was applied to identify underlying patterns among the groups, as shown in **Figure 4A**. The first and second principal components (PCs) accounted for 42.4% and 17% of the total variance, respectively, and the HC group was clearly separated from the disease groups. Six machine-learning models with different learning principles (linear models: Linear Discriminant Analysis and Logistic Regression; nonparametric models: K-Nearest Neighbors and Decision Trees; ensemble model: Random Forest; kernel-based model: Support Vector Machines) were trained with threefold cross-validation to solve the binary classification problems, LN vs. HC and LN-Active vs. LN-Inactive (**Figures 4B, C**). Among them, the Random Forest model outperformed the others in both disease diagnosis (LN vs. HC, AUC = 0.96) and monitoring of LN disease activity (LN-A vs. LN-I, AUC = 0.88). Thus, the Random Forest model was fine-tuned using GridSearchCV on a training dataset comprising 60% (53 samples) of the data for four-group multiclass classification and was tested on the remaining 36 samples. The confusion matrix (**Figure 4D**) shows that the overall macro-average AUC value for the test dataset was 0.94. A small portion of patients with LN-Active were misclassified as having either LN-Inactive or CKD.

### Assessing LN flare status with BPMA-S6

**Figure 5:**
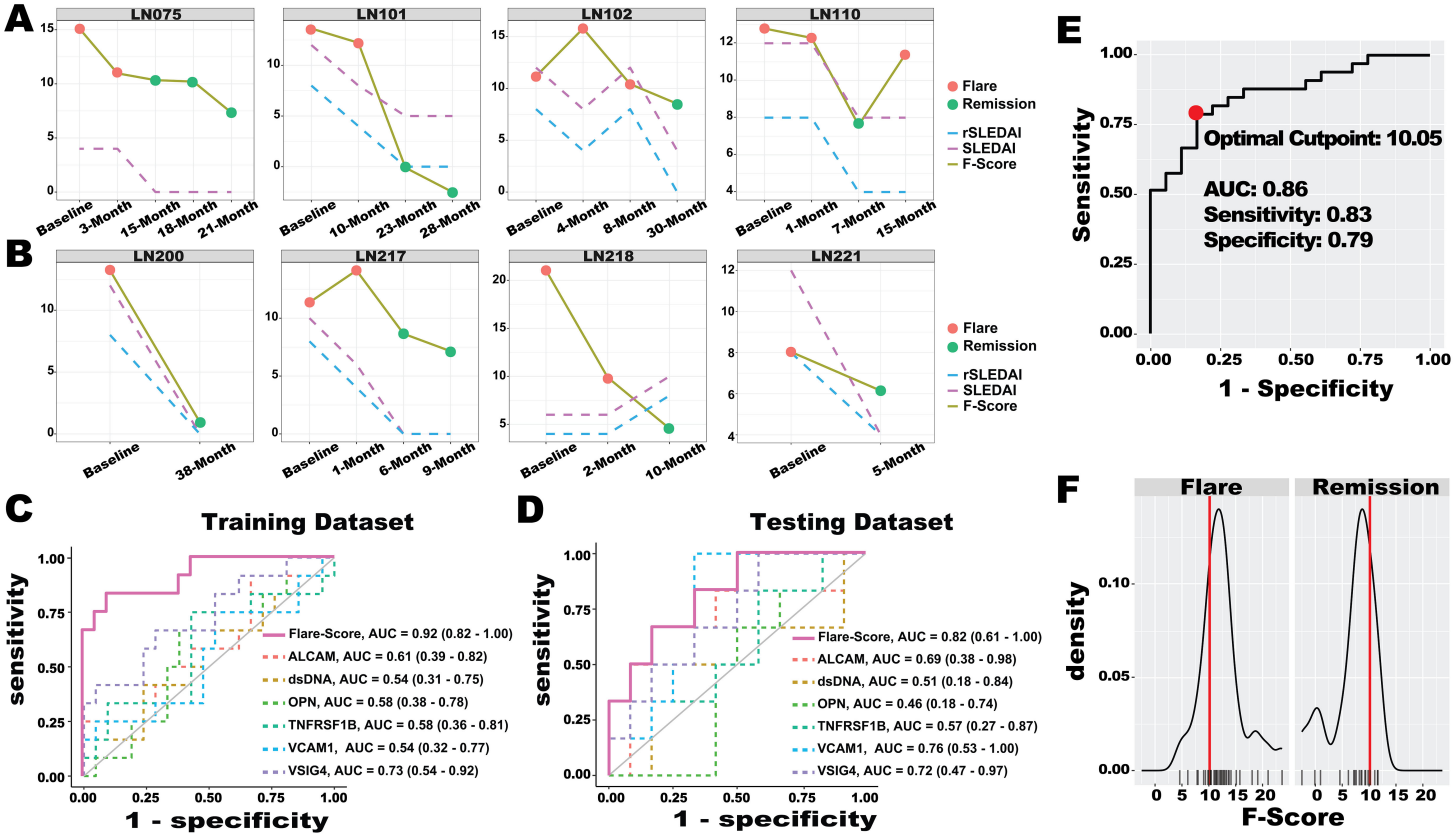
LN flare assessment with ML-based Flare-Score. **(A, B)** Performance of the Flare-Score in tracking disease progression in the training dataset **(A)** and testing dataset **(B)** for four representative LN patients. A total of eight patients with 33 visit samples were used to train the LASSO model to generate the Flare-Score, and seven patients with 18 visit samples were used to test the model. The *x*-axis represents the visiting month, while the *y*-axis shows the Flare-Score and disease activity indexes. Dot colors indicate LN flare status. **(C, D)** The discriminatory abilities of the Flare-Score and BPMA-S6 panel markers in distinguishing LN flare from remission were evaluated in the training dataset **(C)** and testing dataset **(D)**. **(E)** The optimal cut-point for the Flare-Score (red dot, 10.05) was determined based on the highest combined sensitivity and specificity across both datasets. **(F)** Distribution of Flare-Score diagnoses in the flare (left) and remission (right) groups; the red line on the *x*-axis indicates the optimal Flare-Score cut-point (10.05), and the *y*-axis shows the density of flare (left) and remission (right) samples.

We hypothesized that measuring six serum protein biomarkers using BPMA and combining them into a single score could quantitatively and objectively characterize LN flare status, thereby enhancing current LN disease assessment. The BPMA *F*-Score was derived from the weighting matrix of the LASSO model trained on longitudinal LN samples, and its performance was subsequently verified using independent testing samples. As shown in **Figures 5A, B**, the *F*-Score reflected flare and remission status and was correlated with disease activity indexes (SLEDAI and rSLEDAI) in both the training dataset (LN075, LN101, and LN102) and the testing dataset (LN200, LN217, and LN221). For patients LN110, flare status was observed at the last visit, during which the *F*-Score dramatically while the disease activity index remained unchanged. In the case of patient LN218 from the testing dataset, the patient achieved the following two flare episodes—this pattern was consistent with the *F*-Score, whereas the disease active indexes showed an inverse correlation. Notably, the *F*-Score reflected the flare-to-remission transition earlier than both SLEDAI and rSLEDAI in patients LN075, LN110, and LN218, demonstrating the potential of biomarkers for early detection in clinical applications. Compared with the individual biomarkers (**Figures 5C, D**), the *F*-Score demonstrated the highest AUC value in distinguishing flare and remission in the training dataset (AUC = 0.92). As anticipated, the flare-distinguishing AUC (0.82) value dropped in the testing dataset. This is expected. Identifying a specific discrimination value can maximize the success and efficiency of clinical translation (33, 46). As shown in **Figures 5E, F**, the *F*-Score (optimal cut-point: 10.05, AUC = 0.86), which yielded the highest combined specificity (79%) and sensitivity (83%), was selected and validated using all 51 longitudinal LN samples (flare *N* = 33 and remission *N*=18).

## Discussion

Given the heterogeneity of lupus pathogenesis, no individual biomarker can accurately reflect disease activity and its comorbidities (e.g., LN). Different aspects of the pathophysiology or manifestations of lupus or LN may be driven by distinct molecular and signaling pathways. Therefore, identifying key molecules involved in these pathogenic processes is essential for diagnostics, disease monitoring, and therapeutic targeting. Indeed, in recent years, there have been ongoing efforts to identify biomarker panels or composite biomarkers for LN. Proteinuria is an important clinical parameter of LN; however, persistent proteinuria may largely result from preexisting renal damage and may not necessarily indicate ongoing or new renal inflammation or flare during disease progression.

It is worth noting that we are the first to construct a biomarker panel assay on a single chip for the simultaneous detection of five protein biomarkers and anti-dsDNA antibodies to monitor LN disease activity. Mechanistically, the rationale for selecting the six biomarkers in our BPMA-S6 panel includes the following: (1) anti-dsDNA autoantibody is a conventional vital clinical marker of lupus; more importantly, it can form immune complexes with crossreactive antigens, which are subsequently deposited in the kidney parenchyma to initiate lupus nephritis. The synergy of anti-dsDNA antibody-mediated immune complex formation and classical complement activation, along with immune cell infiltration, release of chemokines, cytokines, and proteolytic enzymes, and oxidative damage, can induce kidney inflammation and subsequent organ damage (47); (2) activated leukocyte cell adhesion molecule (ALCAM, also named as CD166) is a cell adhesion glycoprotein expressed on antigen-presenting cells that mediates immune cell adhesion and migration, co-stimulation of T cells, and sustains T-cell activation. It is a ligand of CD6, and the CD6/ALCAM pathway promotes lupus nephritis via T-cell-mediated responses (48). Elevated levels of ALCAM were observed in both serum (this study) and urine (49) of LN patients compared to healthy controls; (3) VCAM-1, a member of the integrin and immunoglobulin superfamily, is induced on endothelial cells in response to inflammatory cytokines, binds to integrin partners on leukocytes, and is elevated in the urine of LN patients (50). Our previous studies showed that urinary VCAM-1 levels were correlated with the renal pathology activity index in LN (51). (4) TNFRSF1B (also known as TNFRII or P75) is a transmembrane receptor for TNF. Its induction has been correlated with the primary site of renal injury (52), and it was found to be elevated in the serum of LN patients (53); (5) osteopontin (OPN) is a pleiotropic protein expressed by various cells. It is upregulated in response to injury and inflammation and plays a role in regulating immune responses (54). Elevated levels of OPN were also observed in the serum of LN patients (53); (6) VSIG4 is a novel transmembrane complement receptor belonging to the immunoglobulin superfamily (also known as CRIg). It functions as an intrinsic inhibitor of complement activation via the alternative pathway. Administration of CRIg-Fc to MRL/lpr mice resulted in reduced kidney inflammation, proteinuria, and pyuria (55). We recently found that VSIG4 was elevated in the serum of LN patients and was associated with renal pathology (Tang et al.).

To date, most biomarker panel studies in LN have focused on urinary biomarkers. For example, transferrin, α1-acid-glycoprotein (AGP), ceruloplasmin, and lipocalin-type prostaglandin D synthetase (L-PGDS) were proposed as a biomarker panel for pediatric LN. The initial evaluation of this panel demonstrated an area under the ROC curve of 0.84-0.88 for assessing LN activity and damage (56). In another study, urinary biomarkers eotaxin 1, GM-CSF, IFN-a2, IFN-g, IL-1a, IL-1b, IL-6, IL-8, IP-10, monocyte chemoattractant protein-1 (MCP-1), MIP-1b, PDGF-BB, lipocalin 2, TWEAK, OPG, cystatin C, and NAG were used to build Random Forest models, which demonstrated a higher AUC and statistical significance compared to models based on traditional clinical markers alone (AUC = 0.79 vs. AUC = 0.61) (57). In a more recent study, individual ELISA measurements of urinary protein biomarkers L-PGDS, ICAM-1, VCAM-1, along with conventional biomarkers anti-dsDNA, C3, and C4, were used to develop a biomarker panel that showed excellent ability to identify renal flare (AUC = 0.98) in a small cohort of LN patients with flare (*N* = 8) (58). In a more recent study, protein biomarkers adiponectin, EGF, and T-cell immunoglobulin mucin-1/kidney injury molecule-1 (TIM-1/KIM1) were combined with baseline clinical parameters—baseline uPCR, baseline eGFR, race, and C4—to construct a biomarker panel using Random Forest modeling. The combined biomarkers were able to reflect treatment responses with an AUC = 0.74 in an internal crossvalidation cohort of LN, in which the protein biomarkers were measured using Luminex custom assay kits (R&D System) and the FlexMap 3D system (Thermo Fisher Scientific) (14). To date, more than 20 proteomic or genomic biomarker panels for lupus have been proposed (13). However, less than 10% of these contain more than 4 biomarkers and the majority of them focused on distinguishing LN from Healthy controls with moderate performance. In comparison, our BPMA-S6 can distinguish LN from HC (AUC = 1) and LN-Active from LN-Inactive (AUC = 0.92). Moreover, our six-plex biomarker pane; demonstrated excellent discriminative value in identifying LN flare in both the training dataset (AUC = 0.93) and testing dataset (AUC = 0.82) within a longitudinal LN cohort.

It is important to note that the six biomarkers in our BPMA-S6 are representative of several major pathogenic pathways of lupus nephritis, including autoantibody production and B-cell activation or migration (anti-dsDNA, VCAM-1, ALCAM) (59–61), T-cell activation (ALCAM, OPN, TNFRSF1B) (48, 62, 63), and complement activation (VSIG4) (55). More importantly, the six biomarkers can be measured simultaneously on a single chip developed in this study, highlighting the great potential of this platform for diagnostics, disease monitoring, or LN flare assessment in clinical practice or clinical trials.

Additionally, we refined the biomarker panel using a LASSO regression model, a machine-learning technique that selects the most relevant features while discarding redundant ones (14, 64). Our recently discovered novel serum biomarker, VSGI4, has demonstrated excellent performance in distinguishing between LN-Active and LN-Inactive nephritis, as well as in reflecting renal pathology activity (Tang et al.), and has significantly contributed to the overall performance of our BPMA-S6.

Similar to paper-based LFAs and vertical flow assays (VFAs), BPMA also offers attractive features such as low cost and portability for clinical use. Importantly, BPMA overcomes limitations seen in LFAs, including issues with multiplexability, the hook effect, and false-negative results. More importantly, BPMA exhibits excellent sensitivity, quantitation capability, and, particularly, multiplexability (65–67). It is worth noting that by optimizing the protocol, we were able to shorten the sample reaction time in the array by > 10-fold—achieving similar detection performance with a 10-min incubation time as with the original 2-h incubation. In addition to using florescent dye Cy3 to detect the array signal, we successfully developed a robust colorimetric-based detection method using SeramunBlau, which simplifies array imaging and eliminates the need for an expensive fluoroimager. This makes BPMA-S6 a more promising platform for implementation in point-of-care settings (68). After optimizing the reaction conditions, the colorimetric detector demonstrated comparable performance to the fluorescence detector in BPMA-S6, including a similar dynamic range of signal detection, sensitivity, specificity, and disease discrimination.

Although our 3D-printed chip cassette and shortened incubation protocol have significantly increased the rapidity of detection, the current protocol for BPMA-S6 assay still includes a washing step. An improved protocol that eliminates the need for washing is desirable. Nevertheless, the BPMA-S6 system may represent a promising serum biomarker panel and detection system for future homecare or community care of LN.

## Data Availability

The original contributions presented in the study are included in the article/supplementary material. Further inquiries can be directed to the corresponding author.
