# Supplementary material for: A serum biomarker panel and miniarray detection system for tracking disease activity and flare risk in lupus nephritis
Source: Front Immunol. 2025 May 1;16:1541907. doi: 10.3389/fimmu.2025.1541907 (PMC12078251; doi:10.3389/fimmu.2025.1541907)
Supplement: Supplementary file 1 [file DataSheet1.pdf]

Supplementary Table S1. ELISA screening of promising LN biomarker candidates

Protein name	Abbreviation	Capture Ab (monoclonal)	Detection Ab (biotinylated)	Results
Galectin-9	LGALS9	R&D Systems	R&D Systems	↑ in LN
Vascular cell adhesion protein 1	VCAM1	R&D Systems	R&D Systems	↑ in LN
Insulin-like growth factor-binding protein 2	IGFBP2	R&D Systems	R&D Systems	↑ in LN
Osteopontin	OPN	R&D Systems	R&D Systems	↑ in LN
Tumor necrosis factor receptor superfamily member 5	CD40	R&D Systems	R&D Systems	↑ in LN
Tumor necrosis factor ligand superfamily member 13B	TNFSF13B/BAFF	R&D Systems	R&D Systems	↑ in LN
CD166 antigen	ALCAM	R&D Systems	R&D Systems	↑ in LN
Platelet factor 4	PF4	R&D Systems	R&D Systems	↑ in LN
Tissue factor pathway inhibitor	TFPI	R&D Systems	R&D Systems	↑ in LN
ADP-ribosyl cyclase/cyclic ADP-ribose hydrolase 2	BST1/CD157	R&D Systems	R&D Systems	↑ in LN
Tumor necrosis factor receptor superfamily member 1B	TNFRSF1B	R&D Systems	R&D Systems	↑ in LN
C-reactive protein	CRP	R&D Systems	R&D Systems	↑ in LN
Monocyte differentiation antigen CD14	CD14	R&D Systems	R&D Systems	↑ in LN
CD177 antigen	CD177	RayBiotech	RayBiotech	↑ in LN
Cystatin-A	CSTA	RayBiotech	RayBiotech	↑ in LN
V-set and immunoglobulin domain-containing protein 4	VSIG4	RayBiotech	RayBiotech	↑ in LN
C-C motif chemokine 21	CCL21	R&D Systems	R&D Systems	NS
Angiopoietin-2	ANGPT2	R&D Systems	R&D Systems	NS
Meprin A subunit beta	MEP1B	R&D Systems	R&D Systems	NS
Pentraxin-related protein PTX3	PTX3	R&D Systems	R&D Systems	NS
Regulator of G-protein signaling 12	RGS12	Santa Cruz	Biorbyt	NS
Hemopexin	HPX	Biorbyt	Biorbyt	NS
Protein phosphatase 1 regulatory subunit 15A	PPP1R15A	Santa Cruz	Biorbyt	NS
Sialoadhesin	SIGLEC1	Biolegend	Biorbyt	NS
Leukocyte immunoglobulin-like receptor subfamily B member 3	LILRB3	Sino Biological	Biorbyt	NS
Guanylate kinase	GUK1	Santa Cruz	Biorbyt	< L-LOD
Properdin	CFP	Santa Cruz	Biorbyt	< L-LOD
Calpastatin	CAST	Santa Cruz	Biorbyt	< L-LOD
Zinc finger protein Aiolos	IKZF3	Santa Cruz	Biorbyt	< L-LOD
Peroxiredoxin-6	PRDX6	Sino Biological	Biorbyt	< L-LOD
Protein S100-A4	S100A4	Biolegend	Biorbyt	< L-LOD
Ubiquitin-conjugating enzyme E2 L3	UBE2L3	Santa Cruz	Biorbyt	< L-LOD

Notes: NS, the difference between LN and HC was not statistically significant; L-LOD, the lowest of the limit of detection.

Supplementary Table S2. Major reagents used in BPMA-S6

Biomarker name	Capture reagent (CR)	Spotting CR (μg/mL)	Composition in Standard-1 mixture (pg/ml)	Detection antibody (biotinylated, dAb)	Cocktail dAb (ng/ml)
dsDNA*	dsDNA	200	/	Goat anti-human IgG, Fcγ fragment specific	1:10,000**
VSIG4	Rabbit anti-human VSIG4	400	800	Goat anti-human VSIG4	100
TNFRSF1B	Mouse anti-human TNFRII	260	4,000	Goat anti-human TNFRSF1B	150
VCAM1	Mouse anti-human VCAM-1	240	90,000	Sheep anti-human VCAM1	200
ALCAM	Mouse anti-human ALCAM	360	13,000	Goat anti-human ALCAM	100
OPN	Mouse anti-human OPN	360	6,000	Goat anti-human OPN	15

*dsDNA was used as a capture reagent.
** A direct dilution from the original product (Cat#109-005-008, Jackson ImmunoResearch Inc).

Supplementary Table S3. Six SLE genomic databases used in this study

Database	GEO Accession	Sample Tissue	Cell Type	# SLE	# HC
Buang_2021	GSE97263	Blood	T CD4+	30	14
Buang_2021	GSE97264	Blood	T CD8+	34	14
Chong_2015	GSE72535	Skin	All celltype RNA extracted	9	8
Berthier_2012	GSE32591	Glomeruli	All celltype RNA extracted	32	14
Berthier_2012	GSE32591	TubuloInterstitium	All celltype RNA extracted	32	15
Akita_2020	GSE156751	Blood	B cell	4	4

Supplementary Table S4. LOD of 5 protein biomarkers on BPMA-S6 using two detectors

Biomarker	Std7 (pg/ml)	Fluorescent		Colorimetric	
		LOD (pg/ml)	4P-Curve R^2	LOD (pg/ml)	4P-Curve R^2
VCAM1	1406.25	299.22	1.00	211.27	0.99
VSIG4	12.50	4.67	1.00	8.09	0.99
ALCAM	203.13	78.24	0.97	120.12	1.00
TNFRSF1B	62.50	48.90	1.00	45.06	0.98
OPN	93.75	27.86	1.00	49.58	1.00

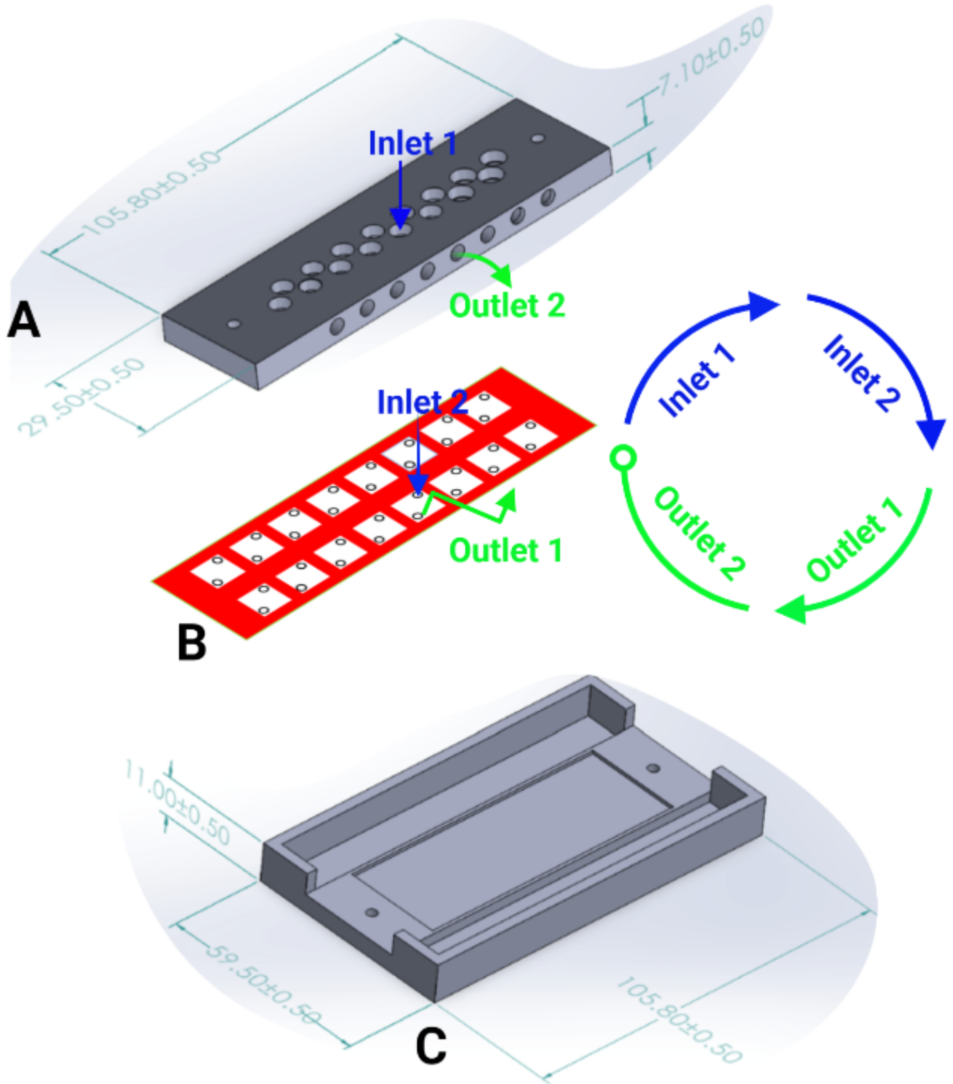

Supplementary Figure S1. A 3D-Printed BPMA chip cassette includes a top part (A), aligning with the chip cover (B) and a tray holder (C). The sample/reagent solution is introduced via Inlet1, flows through Inlet2 to reach the BPMA, and is subsequently discharged through Outlet1 and Outlet2 into a waste tank.

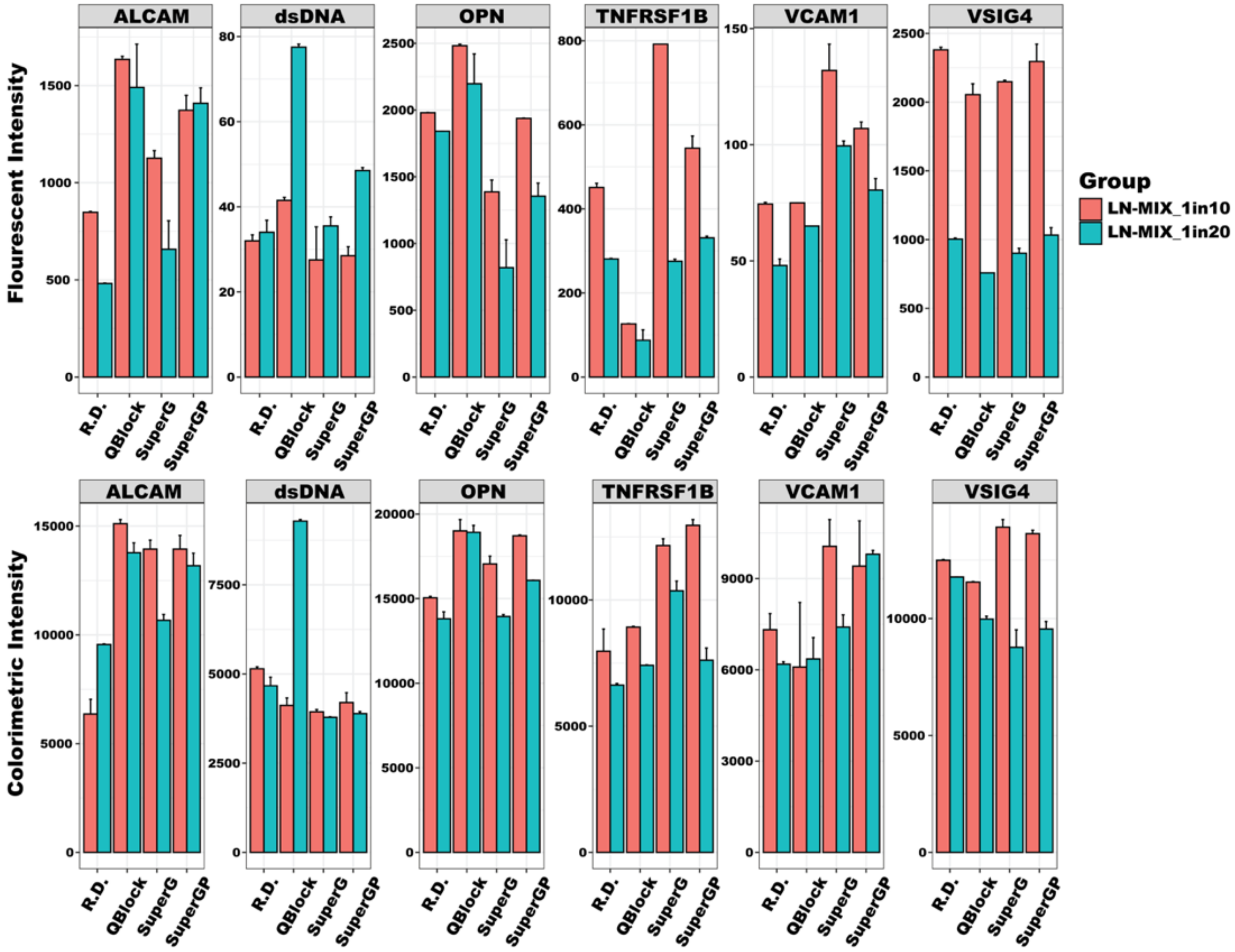

Supplementary Figure S2. Optimization of diluent buffers for the BPMA-S6 assays using clinical samples for Cy3-based fluorescence detection (top) and SeramunBlau-based colorimetric detection (bottom).

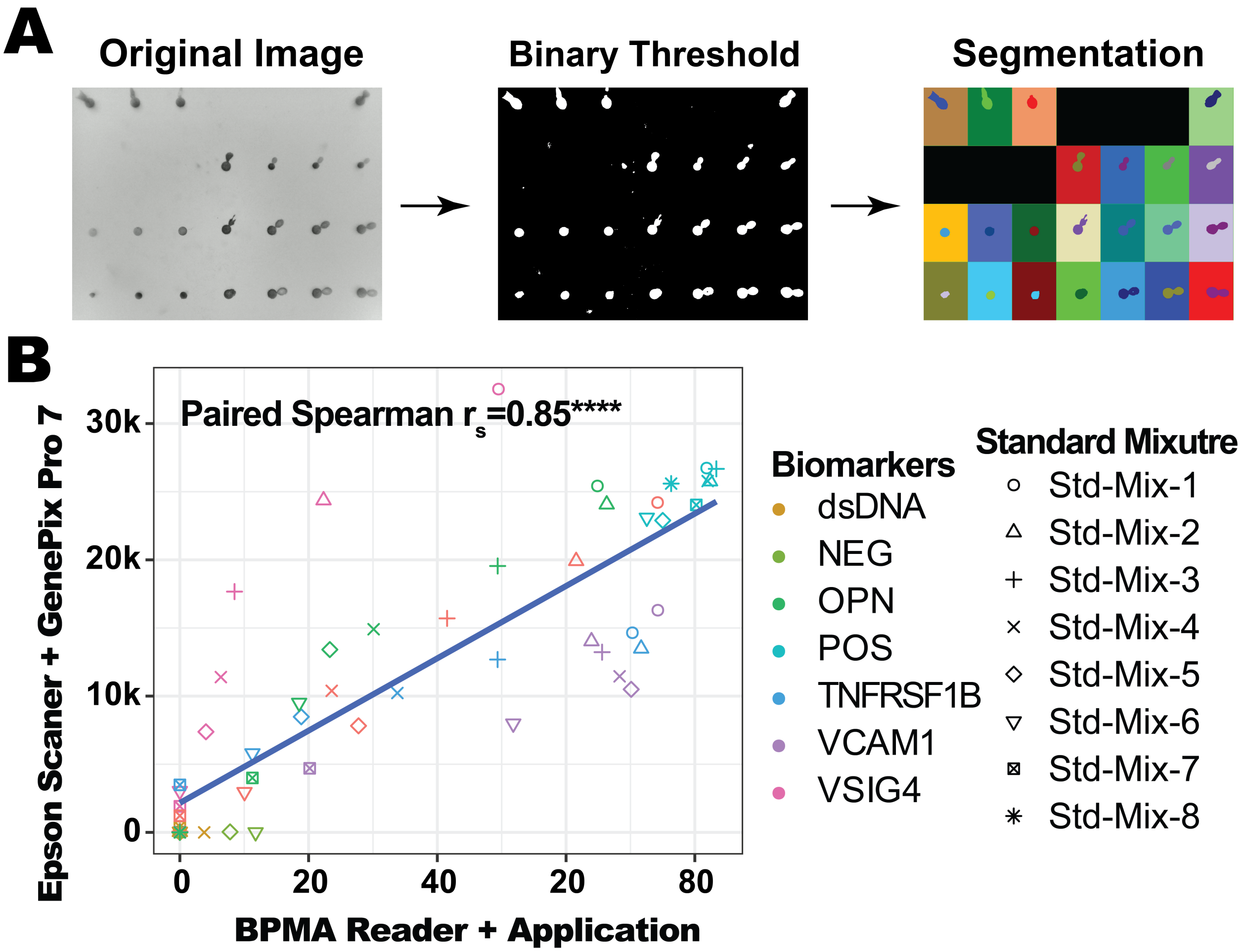

Supplementary Figure S3. Comparison of Commercial scanner and software with BPMA reader and application. (A) The automatic pipeline of image processing on images captured by the BPMA imager and quantified via a smartphone App (B) Spearman's correlation analyses were used to assess their testing correlation.

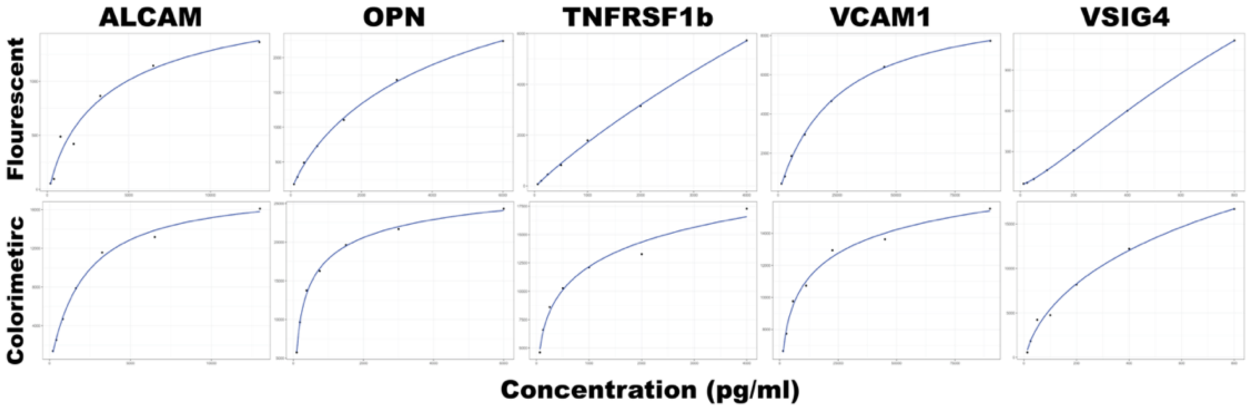

Supplementary Figure S4. Standard Curves of the five protein biomarkers in BPMA-S6.

The 4-parameter logistic (4PL) standard curves were generated for each antigen biomarker from serially diluted mixture standards for Cy3-based fluorescence detection or SeramunBlau-based colorimetric detection.

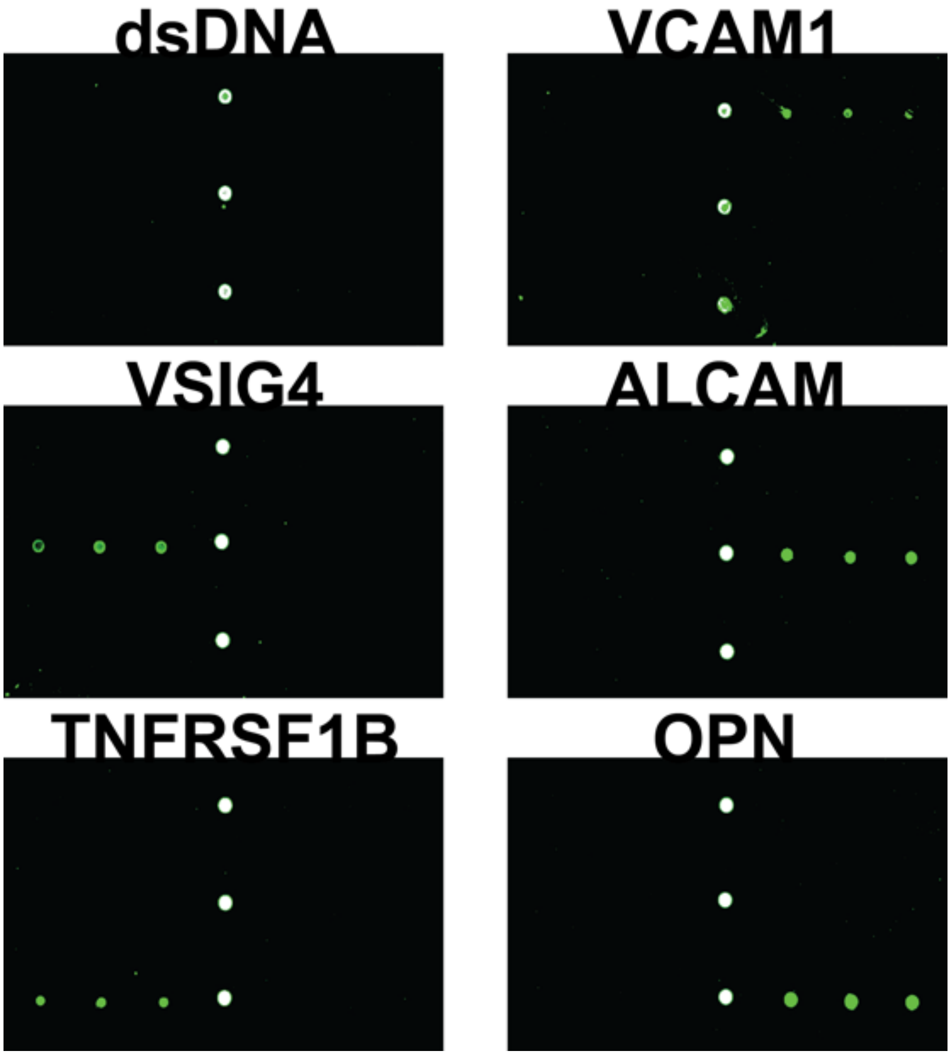

Supplementary Figure S5. The array images of specificity test of BPMA-S6. The pre-coated subarrays with the six biomarker targets were incubated separately with single antigen allocated to each subarray. The vertical three positive-control dots positioned the center of the subarray.

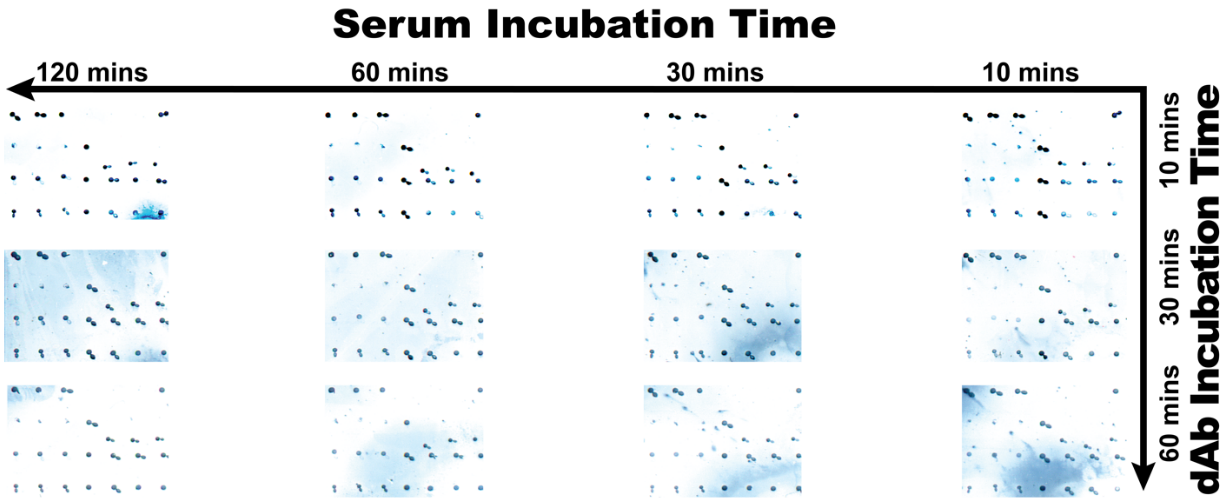

Supplementary Figure S6. The colorimetric detection time evaluation of BPMA-S6 of four serum incubation times (X-axis) and three detection antibody incubation times (Y-axis).

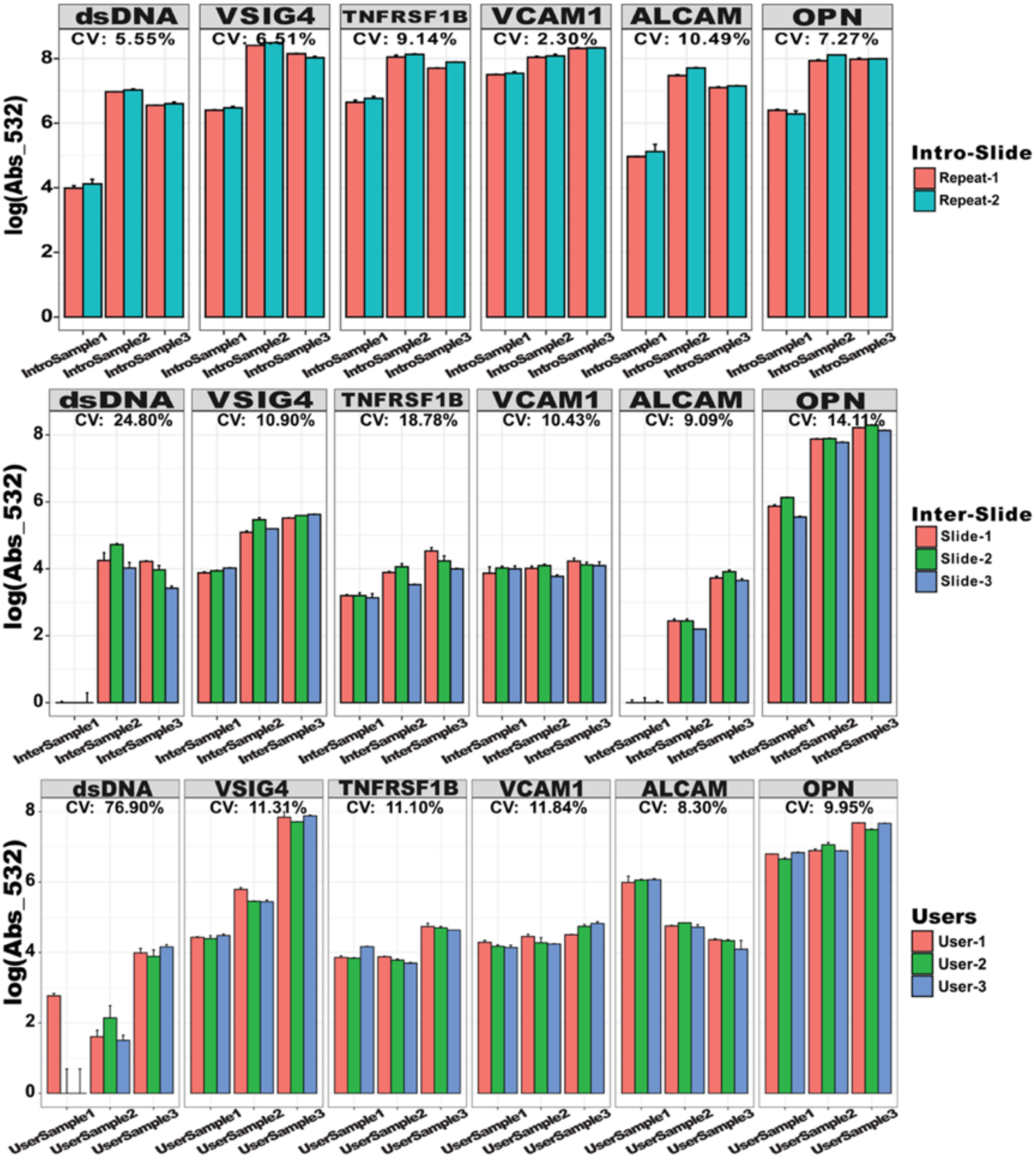

Supplementary Figure S7. Reproducibility tests for BPMA-S6 of Intro-slide (top), Inter-slide (middle), and different users (bottom).

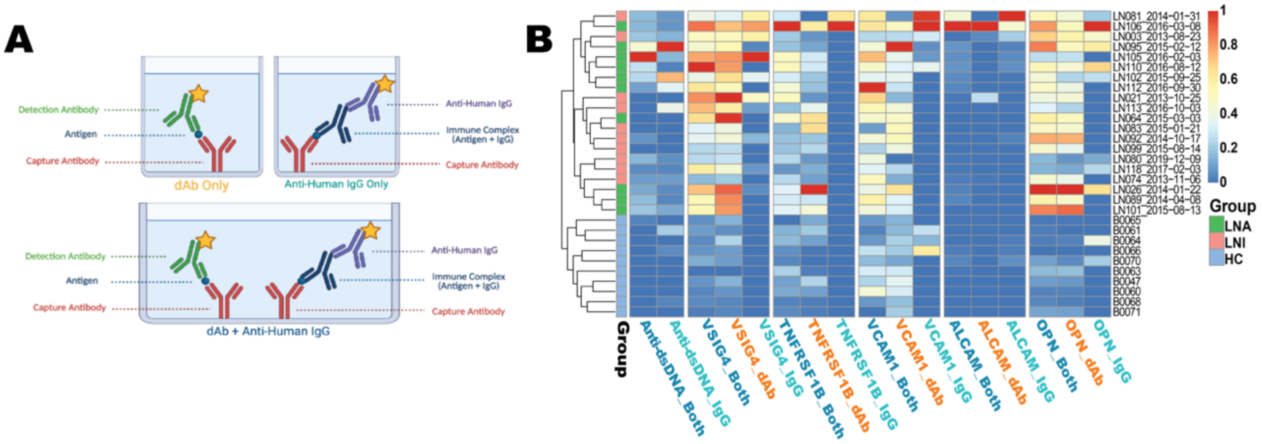

Supplementary Figure S8. Comparison of three types of detection strategies in BPMA-S6. The graphic illustration (A) and 30 serum samples comparison (B) for only five antigen dAbs (dAb-Only, top-left), only anti-human IgG (IgG-Only, top-right), and a full-set cocktail detection solution (both anti-human IgG and five antigen dAb, bottom).

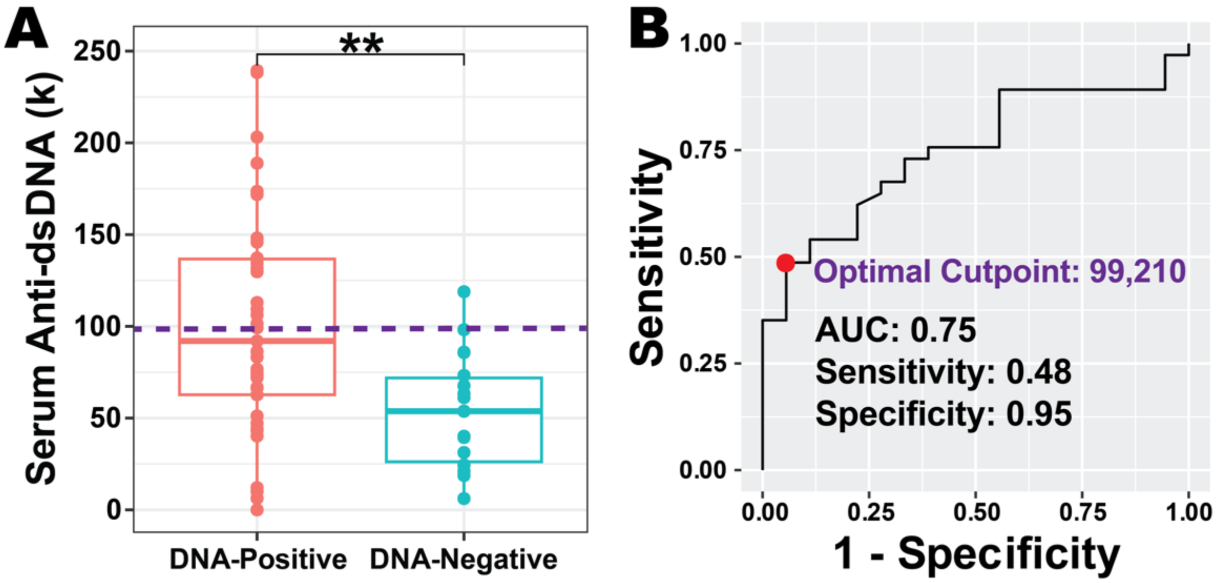

Supplementary Figure S9. BPMA-based anti-dsDNA test could reflect the “positivity” of the clinical test results of anti-dsDNA autoantibody. (A) A lot of 55 serum samples from clinic DNA-Positive (N=37, red) and DNA-Negative (N=18, blue) were tested with BPMA. The purple dash line indicate the chosen cutoff (optimal cutpoint) to distinguish “positivity” and “negative” status. (B) The optimal cut-point for IgG Anti-dsDNA to discriminatory DNA Positive/Negative status. The optimal cut-point (red dot, 99,210) was determined with the highest sum of sensitivity and specificity. Asterisks designate the level of statistical significance: *n.s* $p > 0.05$; *$p < 0.05$; **$p < 0.01$; ***$p < 0.001$, ****$p < 0.0001$.

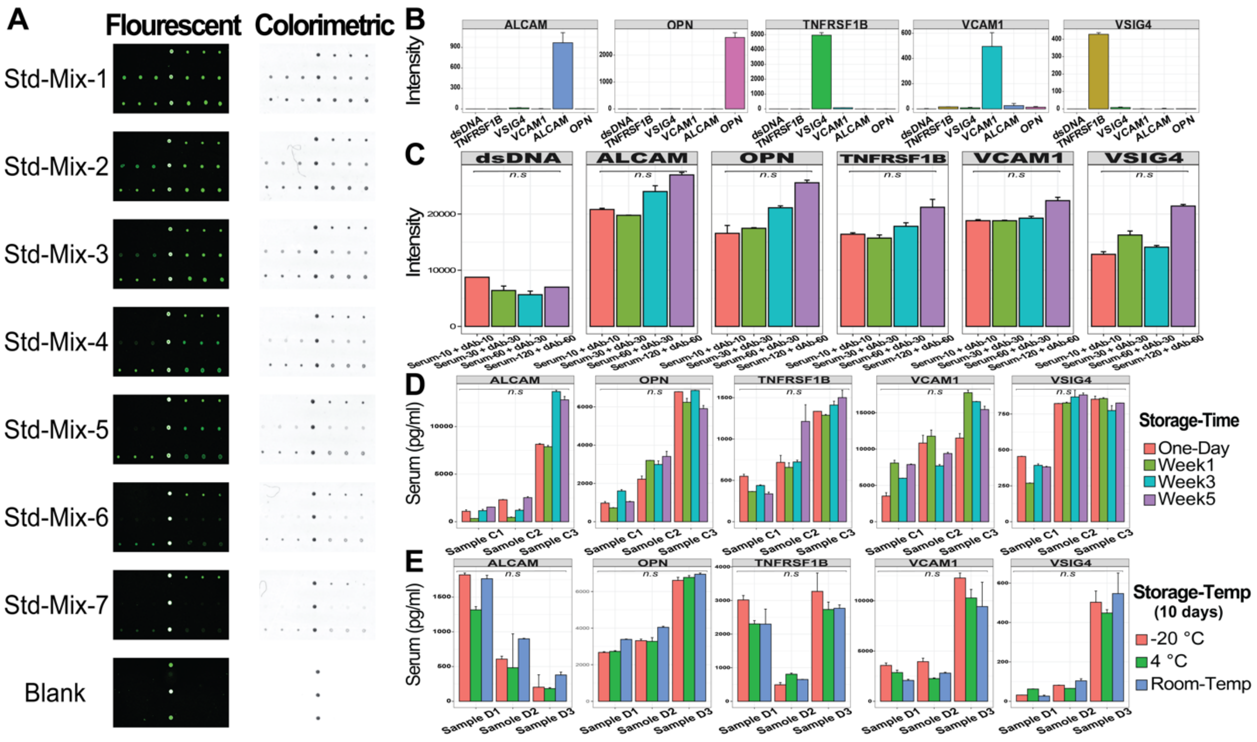

Supplementary Fig S10. Assessment of biomarker panel quantification capability, limit of detection (LOD), cross-reactivity in multiplex, storage stability and thermostability of BPMA-S6. (A) Arrays image of the serial dilution standard mixture. (B) The barplot of BPMA-S6 specificity test. Only one type of antigen was added followed by the cocktail dAb added to each array. (C) The barplot of serum and detective antibody incubation time impact. The different combinations of four serum incubation times and three detection antibody times were individually evaluated. (D) The barplot of storage time impact. BPMA-S6 were packed in vacuum-sealed and desiccant bags at 4°C for one-day, one-week, three-week and five-week to detect three serum samples. (E) The barplot of storage temperature impact. BPMA-S6 were packed in vacuum-sealed and desiccant bags at -20°C, 4°C and room-temperature for 10 days to detect three serum samples. Group significant difference was determined by the Kruskal-Wallis test. Asterisks designate the level of statistical significance: *n.s* $p > 0.05$; *$p < 0.05$; **$p < 0.01$; ***$p < 0.001$, ****$p < 0.0001$.
